# Potential of GC-Combustion-MS as a Powerful and Versatile Nitrogen-Selective Detector in Gas Chromatography

**DOI:** 10.1021/acs.analchem.3c01943

**Published:** 2023-07-25

**Authors:** Javier García-Bellido, Laura Freije-Carrelo, Montserrat Redondo-Velasco, Marco Piparo, Mariosimone Zoccali, Luigi Mondello, Mariella Moldovan, Brice Bouyssiere, Pierre Giusti, Jorge Ruiz Encinar

**Affiliations:** † Department of Physical and Analytical Chemistry, University of Oviedo, 33006 Oviedo, Spain; ‡ TotalEnergies One Tech Belgium, Zone Industrielle C, 7181 Feluy, Belgium; § International Joint Laboratory—iC2MC: Complex Matrices Molecular Characterization, TRTG, 76700 Harfleur, France; ∥ TotalEnergies, TotalEnergies Research & Technology Gonfreville, 76700 Harfleur, France; ⊥ Department of Mathematical and Computer Science, Physical Sciences and Earth Sciences, University of Messina, 98168 Messina, Italy; # Department of Chemical, Biological, Pharmaceutical and Environmental Sciences, University of Messina, 98168 Messina, Italy; ¶ Chromaleont s.r.l., c/o Department of Chemical, Biological, Pharmaceutical and Environmental Sciences, University of Messina, 98168 Messina, Italy; ∇ Universite de Pau et des Pay de l’Adour, E2S UPPA CNRS, IPREM, Institut des Sciences Analytiques et de Physico-chimie pour l’Environnement et les Matériaux UMR5254, 64053 Pau, France

## Abstract

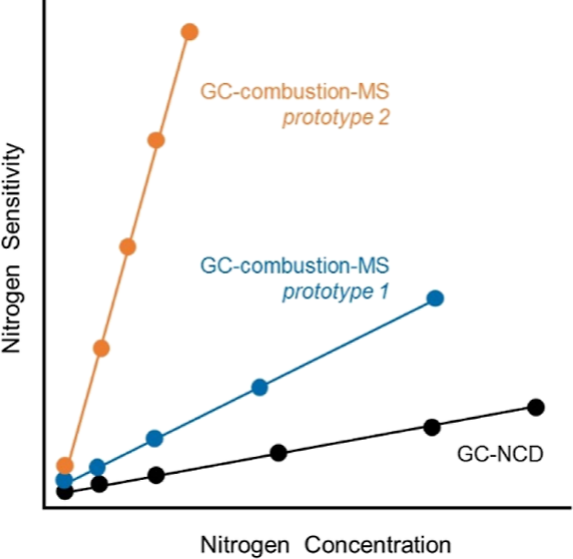

Here, we show the potential and applicability of the novel GC-combustion-MS approach as a nitrogen-selective GC detector. Operating requirements to achieve reproducible and compound-independent formation of volatile NO species as a selective N-signal during the combustion step are described. Specifically, high temperatures (≥1000 °C) and post-column O_2_ flows (0.4 mL min^–1^ of 0.3% O_2_ in He) turned out to be necessary when using a vertical oven without makeup flow (prototype #1). In contrast, the use of a horizontal oven with 1.7 mL min^–1^ He as an additional makeup flow (prototype #2) required milder conditions (850 °C and 0.2 mL min^–1^). A detection limit of 0.02 pg of N injected was achieved, which is by far the lowest ever reported for any GC detector. Equimolarity, linearity, and peak shape were also adequate. Validation of the approach was performed by the analysis of a certified reference material obtaining accurate (2% error) and precise (2% RSD) results. Robustness was tested with the analysis of two complex samples with different matrices (diesel and biomass pyrolysis oil) and N concentration levels. Total N determined after the integration of the whole chromatograms (524 ± 22 and 11,140 ± 330 μg N g^–1^, respectively) was in good agreement with the reference values (497 ± 10 and 11,000 ± 1200 μg N g^–1^, respectively). In contrast, GC-NCD results were lower for the diesel sample (394 ± 42 μg N g^–1^). Quantitative values for the individual and families of N species identified in the real samples by parallel GC–MS and additional GC × GC–MS analyses were also obtained using a single generic internal standard.

The great and continuous development of fuel and biofuel industries, among others, has brought an exponential growth in the number of target compounds to be detected and quantified. In this context, new feedstocks of biological origin or derived from waste (i.e., biomass, landfill waste, and plastics) are renewable and sustainable resources showing high potential to produce fuels and chemicals. Due to their uncontrolled origin, they may contain significant amounts of heteroatomic compounds^1^ that lead to the formation of hazardous components (i.e., NO_*x*_ and SO_*x*_), which can have a negative impact in terms of safety and product quality. Unfortunately, such heteroatoms are split into a myriad of compounds due to the complex character of the chemical processes typically involved in their production (e.g., pyrolysis) and therefore require ultrasensitive and selective approaches for their characterization. In particular, nitrogen-containing compounds have been extensively reported in crude oil-based fuels^2,3^ and other complex matrices such as bio-oil^4^ and plastic-based pyrolysis oil.^5,6^ During refining, N-compounds can cause catalyst poisoning, fouling, equipment corrosion, and gum formation.^7^ For example, N-containing species are harmful for heterogeneous hydrotreatment catalysts during bio-oil pyrolysis, and this adverse effect is species-dependent. This is why it is critical to find out what individual N-species are present, and in which quantity, in order to select and optimize the industrial process for their removal to upgrade the product.^6^ Moreover, their presence in fuels contributes to the environmental release of air pollutants NOx after their combustion.^8^ It is therefore evident the need for analytical technologies able to monitor and quantify N-compounds at ultralow levels in such complex matrices without the need for specific standards due to the huge number of possible targets.

Gas chromatography (GC) is the technique of choice for the separation of volatile compounds that, when coupled with a suitable detector, can accomplish the analysis of a large number of organic compounds in several complex matrices. Given the relevance of N-compounds’ analysis mentioned above, N-selective detectors such as the atomic emission,^9^ nitrogen–phosphorus,^10^ and nitrogen chemiluminescence (NCD)^11,12^ detectors have been developed and are being extensively used in many laboratories. In recent years, the NCD has established as the most powerful N-selective detector due to its selectivity, sensitivity, and equimolarity.^13^ Despite their technical capacities, all these spectroscopic detectors suffer however from some important limitations such as significant matrix^11^ and quenching^14^ effects when analyzing complex unresolved samples. Alternatively, mass spectrometry (MS) provides universal detection and structural identification as well as compound-selective detection when operated in single-ion monitoring (SIM). Unfortunately, this mode is highly limited in complex samples for the screening of specific families of compounds, such as N-containing ones, due to chromatographic coelutions and isobaric interferences in MS.^15^ Additionally, the ionization process in MS is compound-dependent, which entails the need for specific standards to carry out the quantification of every individual N-containing target.^16^

A new detection system in GC able to provide generic carbon-based universal quantification of organic compounds while maintaining the structural elucidation capabilities of MS by simply actuating a switching valve was introduced in 2009.^17^ A combustion interface was developed and installed in a GC–MS instrument for the quantitative conversion of each and every organic compound eluting from the column into CO_2_ before the detection by MS, making their quantification truly compound-independent. Recently, such system was greatly improved based on the idea that the same way as the combustion of an organic at the exit of a GC column produces CO_2_, other volatile species such as H_2_O, SO_*x*_, and NO_*x*_ (if S and N are present) would be produced as well, opening the door to parallel S- and N-selective detection.^18^ Therefore, the long-wished detection combining structural identification with compound-independent calibration, both universal (C, H) and element-selective (N, S), for every volatile organic compound separated by GC became within reach since then.

The focus of this work is to critically assess the potential of the innovative GC-combustion-MS approach as a powerful N-selective detector. We will describe in detail the system optimization and characterization, including the introduction of a new and greatly improved prototype that offers unsurpassed detection limits. Analytical validation will be performed by the analysis of a CRM (carbazole standard). Its analytical figures as a N-selective detector (detection limits, equimolarity, and complementary qualitative information) will be critically compared with those of the established and most widespread GC-NCD, including their applicability to the total and individual quantification of N species without specific standards in complex samples, such as diesel and biomass pyrolysis oil. In this case, the trueness of the results obtained was evaluated in comparison to the corresponding established ASTM methods.

## Experimental Section

### Reagents, Solutions, and Materials

Dichloromethane, hexane, pentadecane (C15), heptadecane (C17), *N*,*N*-diethylaniline (DEA, 99%), *N*,*N*-dibutylaniline (DBA, 99%), quinoleine (Q, 98%), 1-methylindole (1MI, 97%), 2,6-diisopropylaniline (DPA, 100%), indole (I, 99%), 3-methylindole (3MI, 98%), carbazole (C, 95%), 4-ethylpyridine (4EPy, 98%), diethylpropionamide (DEPA, 99%), aniline (A, 99.5%), nitrobenzene (NBz, 99%), 1,2-dimethyl-3-nitrobenzene (DMNBz, 97%), benzonitrile (BZN, 99.9%), caprylonitrilo (HpCN, 99%), and caprolactame (CAP, 100%) (see Figure S1) were purchased from Sigma-Aldrich. The Certified Reference Material D-4629-91-HB-CON, consisting of a nitrogen solution for high boiling solvents containing carbazole (998 μg N g^–1^) in toluene/acetone (9:1), was acquired from AccuStandard. Helium and the mixture of 0.3% (v/v) O_2_ in He were obtained from Air Liquide and Linde AG, respectively. Real samples, diesel and biomass pyrolysis oil, were provided by TotalEnergies Raffinage Chimie.

### Instrumentation

#### GC Separations

Two different analytical columns, a BD-EN14103 (30 m × 0.32 mm ID × 0.25 μm) and a HP1-MS (50 m × 0.2 mm ID × 0.5 μm), both from Agilent J&W Scientific were used. Experimental conditions are summarized in Table S1.

#### GC-Combustion-MS. Prototype #1

Initially, an Agilent 6890 GC coupled with a 5973 Network quadrupole mass spectrometer equipped with an electron ionization source and a split/splitless inlet was used. A manually actuated high-temperature six-way valve (VICI Valco) was installed inside the GC oven to bypass the combustion furnace when necessary, allowing the setup to work under GC–MS (Figure S2A) or GC-combustion-MS (Figure S2B) configurations. The combustion interface, located on top of the GC, consisted of a combustion oven (Carbolite Gero) in which a ceramic tube (400 mm length × 3 mm width × 0.5 mm ID) (Elemental Microanalysis) containing two Pt wires was inserted and connected to the six-way valve by means of a metallic inert tubing and a reducing union (VICI Valco). A flow of O_2_ diluted in He (0.3% v/v) provided by a Mass Flow Controller (MFC, Bronkhorst) was mixed online with the eluting flow from the column before entering the ceramic tube using a capillary flow “Tee” (Capillary Flow inert Tee, Agilent) located inside the GC oven. **Prototype #2**: Later, a much modern instrument (Shimadzu GC-MS-QP-2020NX), equipped with an electron ionization source and a split/splitless inlet, was modified based on the GC-combustion-MS prototype previously described. An automatically actuated high-temperature six-way valve was installed inside the GC oven, allowing the setup to work under GC–MS (Figure S3A) or GC-combustion-MS (Figure S3B) configurations. In this case, the combustion interface was horizontally placed on the right side of the GC oven and connected by means of a metallic block heated at 250 °C, which had two inlets. The first one was used to introduce an additional He makeup flow (ca. 1.7 mL min^–1^) to protect the capillary interface and reduce peak broadening. The second one was used to introduce the O_2_ diluted in He (0.3% v/v). The total flow was then introduced into the same ceramic tube (containing two Pt wires) for combustion.

#### GC-NCD Instrument

The system consisted of a GC Agilent 6890 N equipped with a 255 Agilent NCD detector. A nonpolar HP-1 (50 m × 0.2 mm ID × 0.5 μm) column was used. The system was operated under the optimum conditions indicated by the manufacturer. The combustion temperature was 950 °C with 6.0 mL/min hydrogen and 9.0 mL/min oxygen flow rates.

#### GC × GC–MS Instrument

Additional identification of the N-compounds present in the real samples was carried out using a Shimadzu GC × GC-QqQ MS instrument (operated in SCAN mode), consisting of a GC-2010 with a split/splitless injector and an AOC-20i autosampler, coupled with a TQ8040 MS. SLB-5ms (20 m × 0.18 mm ID × 0.18 μm) and SLB-35 (5 m × 0.32 mm ID × 0.25 μm) columns (Merck Life Science) were used as first and second dimension, respectively. The modulation was performed every 5 s (an accumulation time of 4.6 s and a re-injection period of 0.4 s) by using a flow modulator consisting of a 7-port wafer with an accumulation loop with a dimension of 20 cm × 0.51 μm developed by Chromaleont and Trajan (Trajan Scientific and Medical).^19^ The system was operated in the constant flow mode in both the first and the second dimensions at 0.4 and 8 mL min^–1^, respectively.

## Results and Discussion

### Assessment of the Combustion Efficiency

**Figure 1:**
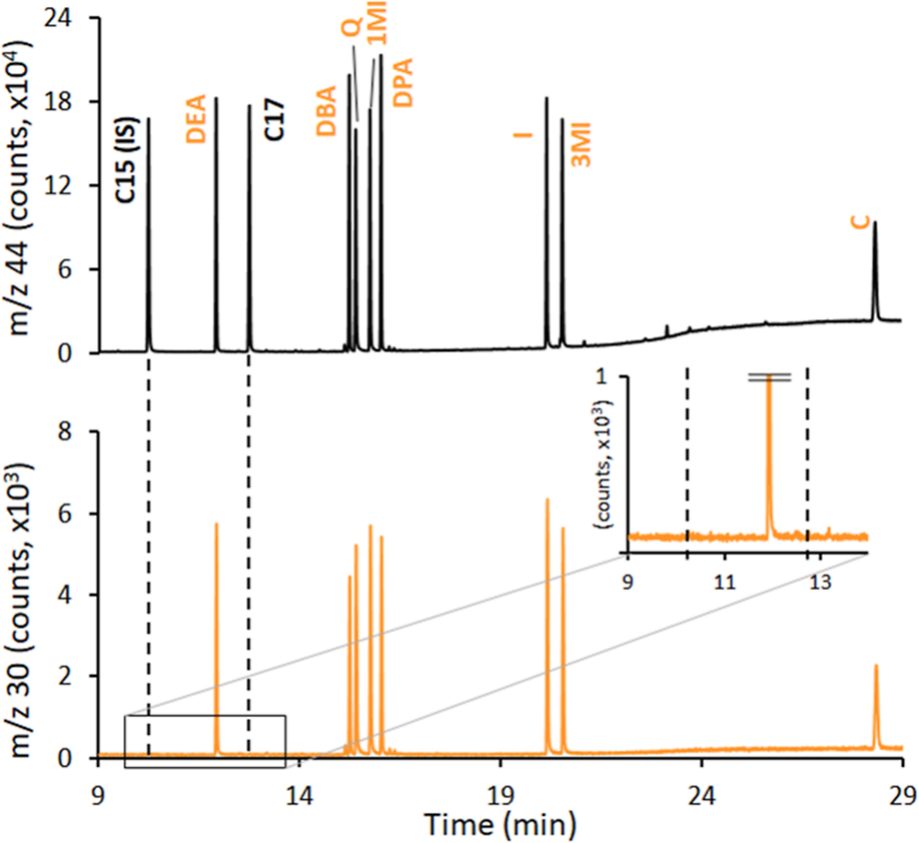
GC-combustion-MS chromatogram of a mixture of eight N-compounds and two alkanes (ca. 5–6 μg C g^–1^ each compound). Compounds’ abbreviations are written out in the Experimental Section. Black and orange lines correspond to *m*/*z* 44 (C) and *m*/*z* 30 (N) signals, respectively. Inset shows the absence of N-signal at the retention times of the two alkanes.

We assessed first the impact of key parameters on the complete combustion of the target compounds using the prototype #1, such as the temperature of the combustion oven and the O_2_/He flow added online post-column. For that purpose, we evaluated the formed ^12^C^16^O_2_ (measured at *m*/*z* 44), originated from the carbon present in each compound. A mixture of eight N-compounds (DEA, DBA, DPA, I, 1MI, 3MI, Q, and C; ca. 5–6 μg C g^–1^) and two alkanes (C15 and C17; ca. 6 μg C g^–1^) was prepared in hexane and injected in triplicate for each condition using splitless mode. Four different temperatures (850, 925, 1000, and 1150 °C) combined with three O_2_/He flows (0.1, 0.2, and 0.4 mL min^–1^) were evaluated. As an example, the black line in Figure 1 shows the GC-combustion-MS chromatogram obtained at *m*/*z* 44 for the eight N-compounds and two alkanes at 1150 °C and 0.4 mL min^–1^. Pentadecane was selected as an internal standard (IS), since its quantitative combustion was already proved,^17^ to compute the recoveries of the rest of the nine eluting peaks of the chromatogram. As can be seen in Figure S4, the average of the nine recoveries computed under each condition assayed was always quantitative, ranging from 96 ± 4 to 99 ± 4% (*n* = 9). These results clearly demonstrated the completeness of the combustion reaction regardless of the temperature and O_2_/He flow used.

### Assessment of the Nitrogen-Selective Detection

**Figure 2:**
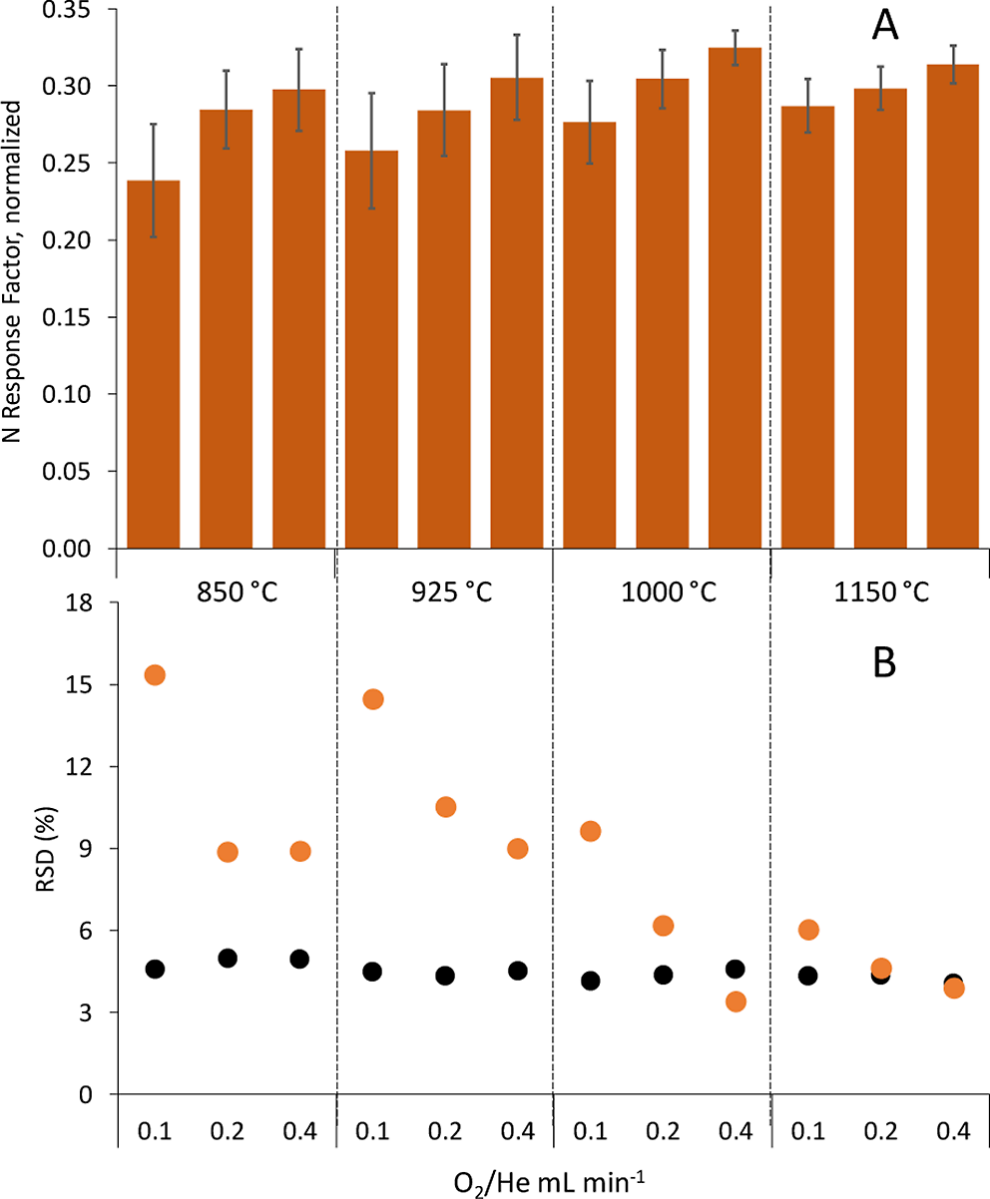
(A) Average N response factor (*n* = 8), expressed as formed NO (*m*/*z* 30) normalized by C response factor at (*m*/*z* 44), obtained at different combustion oven temperatures (850, 925, 1000, and 1150 °C) and O_2_/He flows (0.1, 0.2, and 0.4 mL/min) using the prototype #1. Uncertainty corresponds to one standard deviation (*n* = 3). (B) Relative standard deviation (%) obtained at each temperature and flow for the response factors of C and N (black and orange, respectively).

The previous mixture was also used to assess the potential of the formation of ^14^N^16^O (*m*/*z* 30) as an N-selective signal under different experimental conditions using prototype #1 (orange line in Figure 1). It is difficult to study NO_2_ formation because the ^14^N^16^O_2_ (*m*/*z* 46) signal is highly interfered by the formation of the CO_2_ isotopologue, ^12^C^16^O^18^O (*m*/*z* 46). However, the very similar isotope ratios 44/46 measured in N-compounds (230 ± 3, *n* = 10) and alkanes (235 ± 9, *n* = 10) suggest negligible NO_2_ formation. On the other hand, the absence of signal at *m*/*z* 30 for pentadecane and heptadecane clearly indicates the selective N-detection as NO. The N concentration ranged from 0.45 to 0.82 μg N g^–1^, depending on the species. In order to assess the NO formation under the different instrumental conditions assayed and make it independent of the sensitivity changes intra- and inter-chromatograms performed, we normalized each N response factor (peak area at 30 per N concentration unit) by the corresponding and already demonstrated quantitative C response factor (peak area at 44 per C concentration unit). Figure 2A shows the average of the normalized NO response factors obtained for the eight N-containing compounds at the four different temperatures (850, 925, 1000, and 1150 °C) and three O_2_/He flows (0.1, 0.2, and 0.4 mL min^–1^). It is clear that the observed N response factors increased both with temperature and the O_2_ flow within each temperature. It seems that even though the combustion process is complete already at 850 °C and using the lowest O_2_ flow as indicated by the CO_2_ recoveries (Figure S4), efficient NO formation requires more drastic conditions, what makes sense according to their standard enthalpies of formation at high temperatures (−393 and +90 kJ mol^–1^ for CO_2_ and NO, respectively). In particular, average NO normalized factors were statistically lower (0.24 ± 0.04 and 0.26 ± 0.04) when using the lower O_2_ flow rate at the two lower temperatures. This effect will determine the sensitivity of the N-detection. Notably, while average NO response factors were stable (0.30, 0.31, 0.32, and 0.31) when using the highest O_2_ flow (0.4 mL min^–1^) regardless of the temperature used, their variability increases significantly when moving from higher (3–4% RSD) to lower temperatures (8–9% RSD), as clearly shown in Figure 2B. In fact, the precision of the NO response factors obtained for the eight different N-compounds evolves from values above 15% RSD at 850 and 925 °C and 0.1 mL min^–1^ O_2_ to a plateau around 3–6% RSD obtained at both, 1150 °C regardless the O_2_ flow and 1000 °C only at the highest flows (0.2–0.4 mL min^–1^ O_2_). This effect will determine the equimolarity of the N-detection. In fact, Figures 2B and S4 show that the universal C-detection at *m*/*z* 44 is species-independent always, which suggests that combustion is complete, regardless of the temperature-O_2_ flow combination assayed. In contrast, Figure 2A,B seems to indicate that NO formation is strongly favored, becoming species-independent, only at high (0.4 mL min^–1^) O_2_ flow rates, easing this requirement at higher temperatures. Finally, a temperature ≥1000 °C and a 0.4 mL min^–1^ O_2_/He flow were selected as optimum for N-selective detection when using prototype #1.

**Figure 3:**
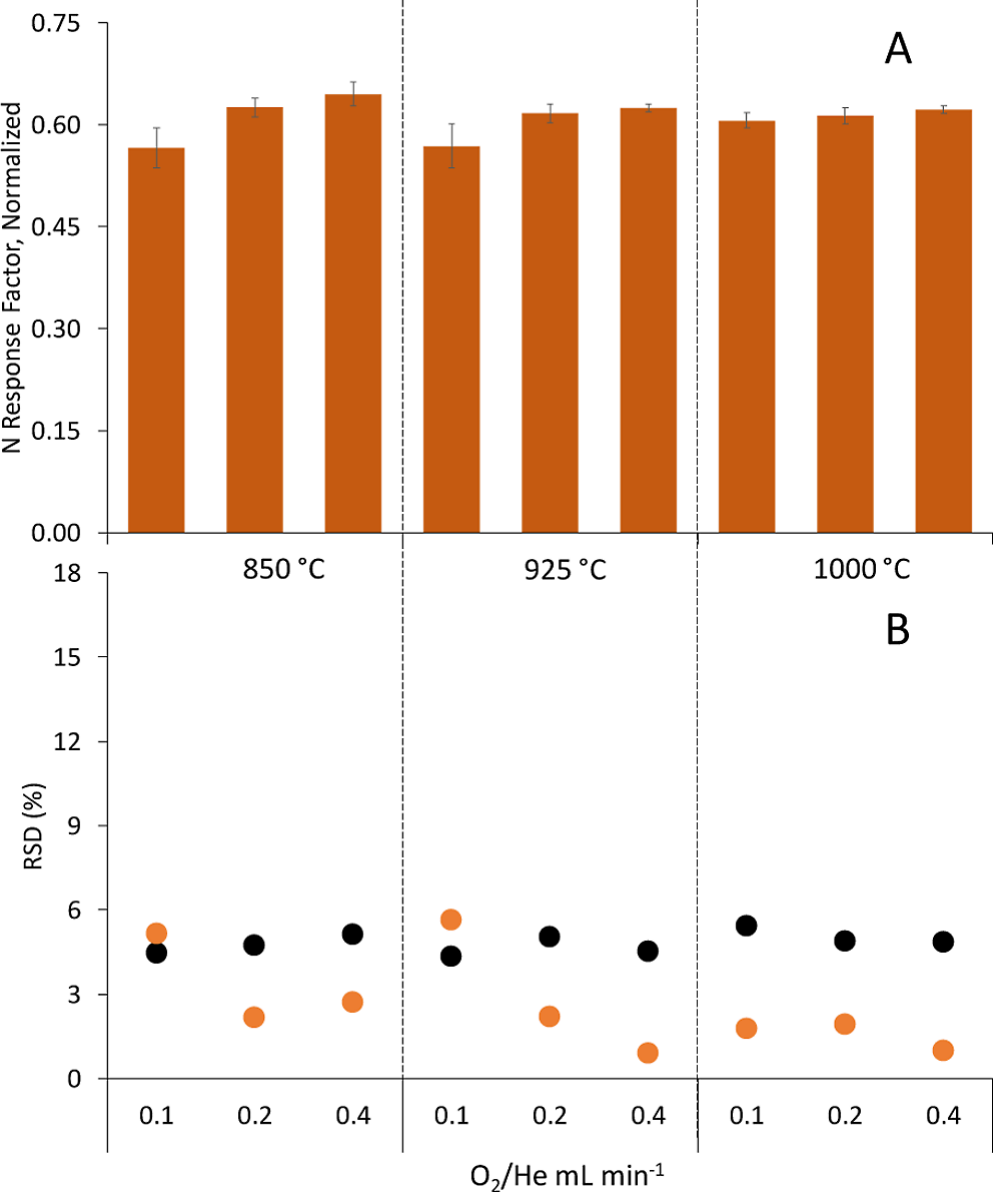
(A) Average N-compounds response factor (*n* = 8), expressed as formed NO (*m*/*z* 30) normalized by C response factor at (*m*/*z* 44), obtained at different combustion oven temperatures (850, 925, and 1000 °C) and O_2_/He flows (0.1, 0.2, and 0.4 mL/min) using the prototype #2. Uncertainty corresponds to one standard deviation (*n* = 3). (B) Relative standard deviation (%) obtained at each temperature and flow for the response factors of C and N (black and orange, respectively).

Surprisingly, the results obtained for the same mixture were quite different when using the prototype #2. As can be seen in Figure S5, the average of the nine carbon recoveries (using pentadecane as reference) computed using the three O_2_/He flows (0.1, 0.2, and 0.4 mL min^–1^) at different temperatures (850, 925, and 1000 °C) was always close to 100% (ranging from 96 ± 5 to 104 ± 4%), indicating again the completeness of the combustion reaction regardless the experimental conditions used. However, the behavior of the average of the eight NO normalized factors was different. Figure 3 clearly shows that, except for the lowest O_2_ flow at 850 (0.57 ± 0.03) and 925 °C (0.57 ± 0.03), the rest of the experimental conditions assayed led to consistent average NO normalized factors with values ranging from 0.61 ± 0.01 to 0.65 ± 0.02. Additionally, the precision of the normalized NO response factors obtained for the eight different N-compounds was always excellent (around 3% RSD) and very similar to the precision of the C response factors, regardless the experimental conditions used. Therefore, it was not necessary to assay higher temperatures (1150 °C) for prototype #2.

**Figure 4:**
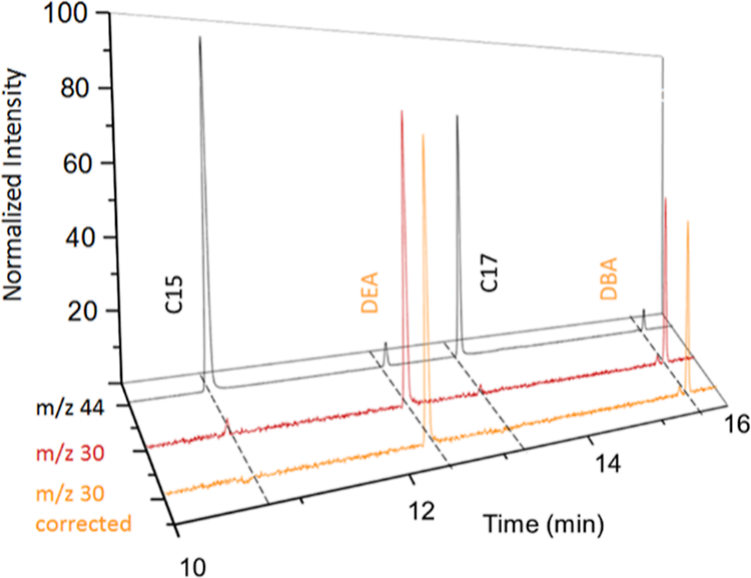
Chromatogram of a mixture of C15 and C17 (ca. 14 and 19 μg C g^–1^, respectively), *N*,*N*-diethylaniline (DEA), and *N*,*N*-dibutylaniline (DBA) (ca. 2 μg C g^–1^ and 0.2 μg N g^–1^): *m*/*z* 44 (black line), *m*/*z* 30 (red line), and corrected *m*/*z* 30 (orange line).

However, the selectivity of the proposed N detection using the NO signal should be evaluated under stringent conditions where the concentration of other organic but noncontaining N-compounds is very high simulating the actual conditions in real complex samples. In this regard, a challenge to the proposed selective N-detection is the in-source fragmentation of CO_2_ to CO. This is generally established and shown in CO_2_ NIST spectra with an abundance close to 10% (28/44 ratio of 0.098). Of course, its very minor isotopologue ^12^C^18^O, detected as *m*/*z* 30, is also produced and could lead to false positives when searching for N-containing compounds using the ^14^N^16^O signal in the sample. The very low abundance of ^18^O (0.2%) makes that this interference is not detected at all in solutions injected at concentrations below 5 μg C g^–1^, as clearly shown in Figure 1. A mixture containing pentadecane and heptadecane at high concentrations (14 and 19 μg C g^–1^, respectively) and two low concentrated N-containing compounds *N*,*N*-diethylaniline and *N*,*N*-dibutylaniline (ca. 2 μg C g^–1^ and 0.2 μg N g^–1^) was injected. As illustrated in Figure 4 (red trace), under these conditions, a tiny but still detectable unspecific signal is observed at *m*/*z* 30 for both alkanes, which therefore requires an appropriate correction. As we can assume that within the ion source, the formation of ^12^C^18^O^+^ (*m*/*z* 30) is isotopically related to the formation of ^12^C^16^O^+^ (*m*/*z* 28), a correction for the unspecific signal at *m*/*z* 30 can be performed based on the ^18^O/^16^O abundance ratio (isotopic abundances of ^16^O and ^18^O are 99.76% and 0.2%, respectively) by measuring in parallel the signal at *m*/*z* 28. In fact, after application of this correction point by point (orange trace), the unspecific *m*/*z* 30 signal for the alkanes is eliminated. Notably, as can be seen in Figure 4, the correction applied did not lead to signal-to-noise ratio deterioration, being the chromatographic profiles of the raw (30) and corrected NO (30/28) very similar. For that reason, we recommend to perform the correction always in the analysis of real samples.

### Analytical Characteristics

First, in order to study peak broadening, a solution containing approximately 2 μg compound g^–1^ of indole and 3-methylindole in *n*-hexane was injected both in the qualitative (GC–MS) and quantitative (GC-combustion-MS) modes. The TIC chromatogram obtained is shown in Figure S6A, whereas the chromatogram obtained at masses 44 (C) and 30 (N) after combustion is shown in Figure S6B. As can be observed, no significant peak broadening due to the combustion unit or to the different connections was observed, being the peak width measured at the half height (0.030 min) only slightly higher to that found when operating the GC–MS in the conventional way (0.025 min). An increase in retention times was observed (∼40 s) because of the combustion furnace.

**Figure 5:**
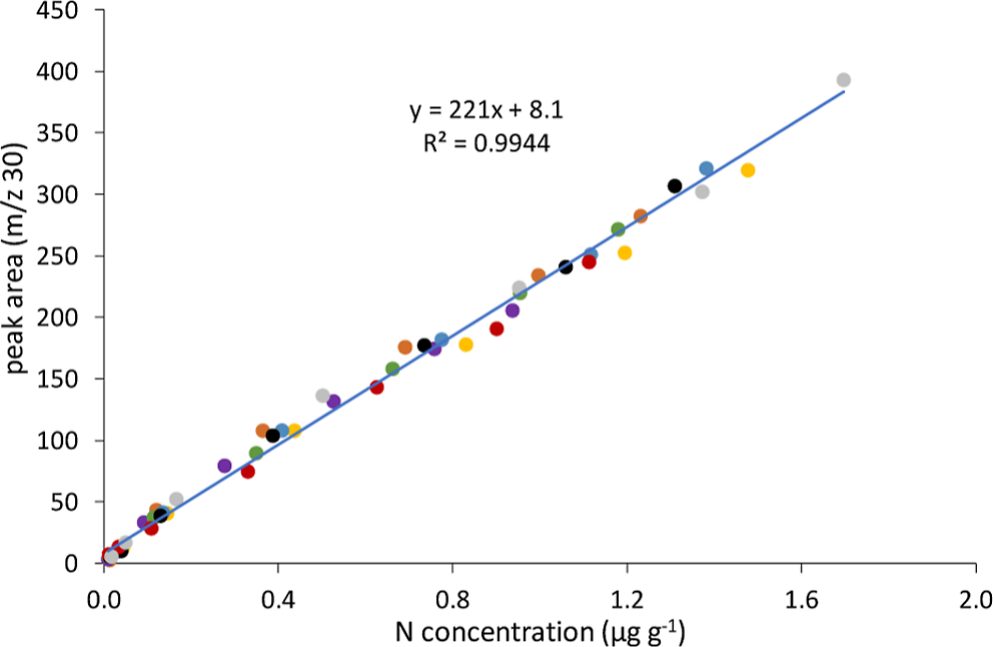
Multispecies generic calibration curve obtained using GC-combustion-MS (prototype #1) for a mixture of N-containing compounds. Color code: *N*,*N*-diethylaniline (orange), indole (grey), 2,6-diisopropylaniline (green), carbazole (red), *N*,*N*-dibutylaniline (violet), 1-methylindole (blue), quinoleine (yellow), and 3-methylindole (black).

Then, in order to study the GC-combustion-MS equimolar response, we resorted again to the same mixture of eight N-compounds covering most of the relevant N-compounds families. Since compound concentration could play a role in the response factors obtained, calibration graphs (*n* = 7) covering more than 2 orders of magnitude (for instance, from 0.016 to 1.7 μg N g^–1^ for indole) were built to check for such concentration-dependent effects. The calibration slopes obtained for every individual N-compound were very similar with excellent linearities. In fact, as shown in Figure 5, a “multispecies” generic calibration plot containing every calibration point of each of the eight N-compounds under study (total *n* = 56) can be built with excellent linearity (*r*^2^ = 0.9944). The most striking conclusion to emerge from Figure 5 is that full species-independent response can be obtained when using GC-combustion-MS as a N-selective detector. This equimolarity adds to that already observed for the GC-combustion-MS system when working as a universal detector through C signal (CO_2_, *m*/*z* 44).^17,20^ Of course, this feature translates into the possibility to carry out accurate quantifications of the diverse N-containing compounds present using a simple N-containing compound as an internal standard. Such possibility was assessed with an extended mixture containing up to 16 different N-compounds including different new N-functionalities such as open and closed amides (*N*,*N*,-diethylpropionamide and caprolactam), pyridines, nitriles (caprylo and benzonitrile), and nitro derivates. In order to avoid coelutions, the different N-compounds were distributed in three different mixtures and 2,6-diisopropylaniline was chosen always as an internal standard. Figure S7 displays the different chromatograms obtained, while quantitative results are given in Table S2. As can be observed, this methodology allowed us to quantify a full series of N-compounds with a broad range of boiling points (from 168 to 355 °C), with acceptable precision (an average value of 2% RSD, *n* = 3) and accuracy (a mean recovery of 98 ± 5%, 2 SD, *n* = 15), without the need for specific standards. For comparison purposes, we wanted to evaluate the equimolar response of the N-selective detector of reference, GC-NCD by analyzing a mixture of N-compounds covering different organic chemical structures and in a range of concentrations from ca. 4 to 100 μg N g^–1^. Such higher concentration range was selected due to the much lower sensitivity observed of the GC-NCD compared to the GC-combustion-MS. As it can be seen in Figure S8, a “multispecies” generic calibration plot (total *n* = 42) could be built with excellent linearity (*r*^2^ = 0.994) as well.

Detection limit (DL) was then calculated for the prototype #1 based on three times the standard deviation of the baseline and turned out to be 0.7 pg of N injected. This is already better than the DL of the well-established N-selective GC-NCD instrument (typically ranging from 2 to 20 pg N).^21,22^ Nevertheless, the great improvement was observed when using the prototype #2, which provided an impressive DL of 0.02 pg of N injected, at least 2 orders of magnitude lower than NCD and, to the best of our knowledge, by far the lowest detection limit for N ever published for a GC detector. Such much higher sensitivity is borne out by the calibration plots obtained for indole using prototype #2 (0.0013 to 0.13 μg N g^–1^), prototype #1 (0.02 to 1.7 μg N g^–1^), and NCD (3 to 84 μg N g^–1^) as shown in Figure S9. It must be noted that the great sensitivity improvement in N-detection using the prototype #2 in comparison to the prototype #1 is most likely due to the brut sensitivity difference between a modern (#2) and an old (#1) MS instrument.

### Validation

A certified reference material, carbazole (998 ± 24 μg N mL^–1^) was spiked with 3-methylindole as an internal standard and analyzed by GC-combustion-MS. Figure S10 shows the chromatogram obtained. Experimental results obtained, 977 ± 39 μg N mL^–1^ (uncertainty corresponds to 95% confidence interval, *n* = 5), was within the 95% confidence interval of the CRM. In addition, precision was as low as 2% RSD. Such results validate our approach and demonstrate its potential for the accurate and precise quantification of N-containing compounds using a single generic standard.

### Analysis of Real Samples

**Figure 6:**
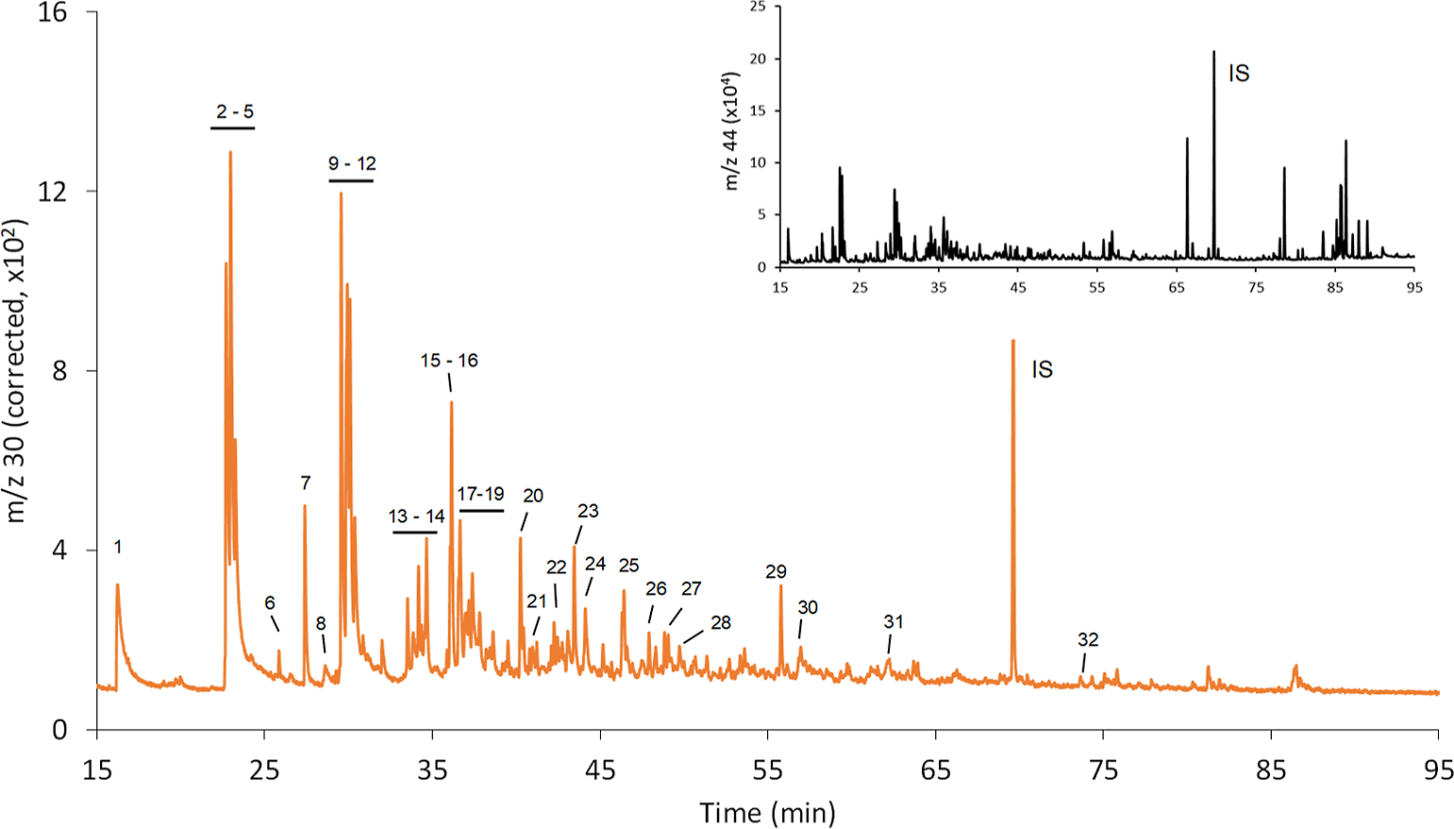
N-selective (*m*/*z* 30 corrected, orange line) and universal (C, *m*/*z* 44, inset, black line) GC-combustion-MS analysis of biomass-based pyrolysis oil using *N*,*N*-diethylaniline as an internal standard (IS). Identification and individual absolute quantification (μg N g^–1^) of the numbered chromatographic peaks are listed in Table 1.

Finally, the applicability of the proposed approach to real sample analysis was tested with two different matrices, a biomass pyrolysis oil and a diesel. The biomass pyrolysis oil was diluted 1:300 in hexane and spiked with *N*,*N*-diethylaniline as an internal standard. Figure 6 presents the N-selective (corrected *m*/*z* 30) GC-combustion-MS profile, while its inset shows the universal (carbon, *m*/*z* 44) profile that matches pretty well with the universal GC–MS profile given in Figure S11. Most of the N-containing peaks were detected at the beginning of the gradient (20–55 min) and corresponded to low abundant compounds in the sample as clearly shown in the inset of Figure 6 where most abundant organic compounds eluted later (65–90 min). The correction using the signal at *m*/*z* 28 was applied, although no significant differences were observed between the raw and corrected as the C concentration of the most intense peaks was below 7 μg C g^–1^. The total N content obtained after integration of the whole N chromatogram and using response factor of the spiked *N*,*N*-diethylaniline was found to be 11,140 ± 330 μg N g^–1^ (2 SD, *n* = 3), which is statistically undistinguishable from the reference value obtained using the well-established total chemiluminescence detection (11,000 ± 1200 μg N g^–1^, similar to the ASTM 4629 method), and very similar to the GC-NCD results (10,640 ± 320 μg N g^–1^, Figure S12). The selective N-chromatographic profile obtained was then used to help in the identification (MS similarity, NIST library) of the different N-containing species present using first the GC–MS configuration of our system. However, due to the sample complexity, only few compounds (16) could be identified. We resorted then to the resolving power of the multidimensional GC × GC–MS to boost species identification. For that purpose, we first used the 16 N-compounds previously detected and identified by our approach to establish a correlation between the retention times in the GC-combustion-MS and GC × GC-MS instruments that could be later used to translate the identification of another 16 compounds achieved by GC × GC-MS to the GC-combustion-MS chromatogram. The result of this multitechnique approach was the identification and quantification of up to 32 N-containing compounds in the complex biomass pyrolysis oil sample. Table 1 summarizes the qualitative and quantitative information obtained to provide an in-depth quantitative characterization of the N speciation in the biomass pyrolysis oil. The sum of the successful individual quantifications accounted for 70% of the total N quantified in the sample because some N-compounds could not be reliably identified.

**Table 1:** List of N-Containing Compounds Identified in the Biomass-Based Pyrolysis Oil (Figure 6)^a^

peak	compound	μg N g^–^^1^
1^b^	methylpyrazine	593 ± 78
2^b^	2,6-dimethylpyrazine	2346 ± 76
3	4,6-dimethylpyrimidine	
4	5-methyl-2-pyridinamine	
5^b^	2-ethylpyrazine	
6^b^	1-ethylpyrrole	22 ± 6
7^b^	isopropylpyrazine	213 ± 28
8	aniline	49 ± 8
9^b^	2-ethyl-6-methylpyrazine	2219 ± 366
10^b^	2-ethyl-5-methylpyrazine	
11^b^	2-ethyl-3-methylpyrazine	
12^b^	Propylpyrazine	
13^b^	*N*-methylsuccinimide	568 ± 64
14^b^	1-pentylpyrrole	
15^b^	1-ethyl-2,5-dimethylpyrazine	352 ± 56
16	2,6-diethylpyrazine	
17^b^	2-methyl-5-propylpyrazine	164 ± 12
18	*N*-ethylsuccinimide	
19	2-isobutyl-3-methylpyrazine	
20^b^	5*H*-5-methyl-6,7-dihydrocyclopentapyrazine	167 ± 54
21	3,5-diethyl-2-methylpyrazine	34 ± 8
22	2,5-dimethyl-3-propylpyrazine	61 ± 6
23^b^	2-pentylpyrazine	174 ± 32
24^b^	2-methyl-5-trans-propenylpyrazine	145 ± 38
25	5*H*-2,5-dimethyl-6,7-dihydrocyclopentapyrazine	164 ± 30
26	3-ethyl-4-methyl-1*H*-Pyrrole-2,5-dione	53 ± 22
27	2-butyl-3-methylpyrazine	52 ± 16
28	2-methyl-3-propylpyrazine	57 ± 12
29	3-methylquinoline	90 ± 22
30	5-methylindole	86 ± 44
31	1-(2′-phenylethyl)-pyrrole	64 ± 2
32	5,6,7-trimethyl-1*H*-indole	16 ± 2

a Individual compound independent quantification was carried out using *N*,*N*-diethylaniline as an internal standard. Uncertainty corresponds to two standard deviations (*n* = 3).

b Identified using the GC–MS mode. Rest of the peaks were identified by GC × GC–MS.

Next, in order to validate further our approach with a more challenging real sample, we selected a diesel with much lower total N content, 497 ± 10 μg g^–1^ (95% confidence level, previously determined by the well-established total chemiluminescence detection). It was injected after a dilution of ca. 1:450 in hexane (Figure S13). Despite the low total nitrogen injected (ca. 1 μg N g^–1^), N-selective detection unveiled many N-containing compounds. Due to the much more complex matrix and much lower concentration of the N-compounds, correction of the *m*/*z* 30 signal was mandatory. In fact, elution of highly concentrated (>10 μg C g^–1^) paraffin species at the beginning of the gradient (19–30 min, clearly shown in the *m*/*z* 44 profile given in the inset to Figure S13A and in the GC–MS profile given in Figure S14), resulted in significant interference signals at *m*/*z* 30 (Figure S13A) due to the already commented ^12^C^18^O formation in the ion source. However, after application of the isotopic correction using the signal at *m*/*z* 28 (^12^C^16^O), such interference peaks were turned out negligible and the corrected N-profile shown in Figure S13B became dominated by the elution of the major families of N compounds at longer elution times (indoles at 32–37 min and carbazoles at 45–50 min). *N*,*N*-Diethylaniline was spiked as an internal standard, and the total N obtained (524 ± 22 μg N g^–1^, 2 SD, *n* = 3) was again statistically undistinguishable from the reference value. The concentrations of the major families of N compounds are given in Table S3. For further internal validation purposes, the diesel sample was also injected in a longer (50 m) and more apolar (HP-1) column using another internal standard (Quinoleine) and the total N obtained (493 ± 47 μg N g^–1^, 2 SD, *n* = 3) was again in excellent agreement. Interestingly, the GC-NCD (Figure S15) results obtained in this case (394 ± 42 μg N g^–1^, 2 SD, *n* = 3) were significantly lower than both the reference value (ASTM 4629 method) and our approach, likely due to the significant and well-known quenching effects suffered by this spectroscopic detector when analyzing complex samples.^11,14^

## Conclusions

This work describes in detail the capabilities of the GC-combustion-MS approach as a powerful and unique nitrogen selective detector. We have herein demonstrated that every N-containing species produces ^14^N^16^O that can be used for their highly selective and sensitive detection and species-independent quantification. The successive and improved prototypes presented and evaluated offer several advantages over established N-selective detectors. First, the ultimate GC-combustion-MS prototype provides by far the lowest detection limit ever reported for N-containing species (0.02 pg N injected). Second, the additional detection at *m*/*z* 44 of the CO_2_ generated for any C-containing compound provides simultaneous universal and compound-independent detection. Third, the structural elucidation capabilities of conventional EI-MS are maintained intact and available after a parallel injection in the same instrument. Fourth, the system showed excellent robustness and reliability in the analysis of two real samples (biomass pyrolysis oil and diesel) with different matrix complexity and N concentration level.

We have proved that the N-selective profile greatly helps in digging into the MS data in the search for the identity of the N-species detected. However, such improved identification capabilities are still severely constrained in complex samples, which obliged us to resort to parallel multidimensional GC × GC–MS analysis to expand the list of N-compounds individually quantified. In fact, future implementation of the proposed instrumental setup in a standard GC × GC–MS instrument could bring together in the same instrument the excellent quantitative (universal, C and element-selective, N) features and powerful identification capabilities of both approaches.

Applications can be foreseen in a wide variety of fields from the petroleum and chemical (polymer, plastic) industries to quantitative metabolomics where the determination of the great and rising variety of N-containing compounds is increasingly important. Finally, it is worth mentioning that the element-selective detection capabilities of the GC-combustion-MS setup is not limited to N-compounds but could also be applied to S-containing compounds through the measurement of the volatile SO_*x*_ species generated after combustion. Of course, such complementary information (still not investigated in detail) would extend even further its potential niche applications.

## Supporting Information Available

List of compounds used, schemes of both prototypes, recoveries and reproducibility studies, chromatograms of mixtures of N-compounds, calibration curves, GC–MS, GC-NCD and GC-combustion-MS chromatograms of samples, chromatographic conditions, and quantitative results of mixtures and real samples (PDF).
