# Supplementary material for: Potential of GC-Combustion-MS as a Powerful and Versatile Nitrogen-Selective Detector in Gas Chromatography
Source: Anal Chem. 2023 Jul 25;95(31):11761–8. doi: 10.1021/acs.analchem.3c01943 (PMC10413323; doi:10.1021/acs.analchem.3c01943)
Supplement: Supplementary file 1 — ac3c01943_si_001.pdf [file ac3c01943_si_001.pdf]

Supporting Information

Potential of GC-Combustion-MS as powerful and versatile Nitrogen Selective Detector in Gas Chromatography

Javier García-Bellido,[a,‡] Laura Freije-Carrelo,[b,c,‡] Montserrat Redondo-Velasco,[a] Marco Piparo,[c,d] Mariosimone Zoccali,[e] Luigi Mondello,[f,g] Mariella Moldovan,[a] Brice Bouyssiere,[ch] Pierre Giusti,[c,d] and Jorge Ruiz Encinar[a]*

[a] Department of Physical and Analytical Chemistry, University of Oviedo, 33006, Oviedo, Spain

[b] TotalEnergies One Tech Belgium, Zone Industrielle C, 7181 Feluy, Belgium

[c] International Joint Laboratory – iC2MC: Complex Matrices Molecular Characterization, TRTG, 76700 Harfleur, France

[d] TotalEnergies, TotalEnergies Research& Technology Gonfreville, 76700 Harfleur, France

[e] Department of Mathematical and Computer Science, Physical Sciences and Earth Sciences, University of Messina, 98168 Messina, Italy

[f] Department of Chemical, Biological, Pharmaceutical and Environmental Sciences, University of Messina, 98168 Messina, Italy

[g] Chromaleont s.r.l., c/o Department of Chemical, Biological, Pharmaceutical and Environmental Sciences, University of Messina, 98168 Messina, Italy

[h] Universite de Pau et des Pay de l'Adour, E2S UPPA CNRS, IPREM, Institut des Sciences Analytiques et de Physico-chimie pour l'Environnement et les Matériaux UMR5254, 64053 Pau, France

* Jorge Ruiz Encinar ruizjorge@uniovi.es

Table of contents

Figure S1. List, chemical structure, and purity of the N-containing compounds used ………..S3

Figure S2. Scheme of the six-way valve and its connections within GC-combustion-MS **prototype #1**. ……………………………………………..………………..…………S4

Figure S3. Scheme of the six-way valve and its connections within GC-combustion-MS **prototype #2**……………………………………………………………………………S5

Figure S4. Average of the recoveries for heptadecane (C17) and eight N-containing compounds (DEA, DBA, Q, 1MI, DPA, I, 3MI, see Figure S1) obtained from the CO_2 signals (m/z 44) under different combustion oven temperatures (850, 925, 100 and 1150 °C) and O_2/He flows (0.1, 0.2 and 0.4 mL min^{-1}) and using prototype #1 and pentadecane (C15) as internal standard…..……S6

Figure S5. Average of the recoveries for heptadecane (C17) and eight N-containing compounds (DEA, DBA, Q, 1MI, DPA, I, 3MI, see Figure S1) obtained from the CO2 signals (m/z 44) under different combustion oven temperatures (850, 925, and 1000 °C) and O_2/He flows (0.1, 0.2 and 0.4 mL min^{-1}) and using prototype #2 and pentadecane (C15) as internal standard……..………S6

Figure S6. Peak shape comparison of two N-containing compounds, indole (2.1 µg compound/g; 1.7 µg C/g; 0.25 µg N/g) and 3-methylindole (2.2 µg compound/g; 1.8 µg C/g; 0.23 µg N/g) analyzed under: **(A)** GC-MS mode; **(B)** GC-combustion-MS mode with universal C detection (m/z 44, black) and N-selective detection (m/z 30, orange)……………………………………..S7

Figure S7. GC-combustion-MS chromatograms of the extended mixtures of nitrogen-containing compounds. ……………………………………………………………………………..S8

Figure S8. Multispecies generic calibration curve obtained using GC-NCD for six N-compounds in a range of concentrations up to 90 µg N g^{-1}..…………………………………..……..S9

Figure S9. A) Calibration plots obtained for indole using prototype #1 (blue) and #2 (orange). 25-fold increase in sensitivity (slope) was observed for prototype #2. **B)** Calibration plots obtained for indole using prototype #2 (orange) and GC-NCD (black)………………...……S10

Figure S10. GC-combustion-MS chromatogram of the aliquot of the CRM D-4629-91-HB-CON (carbazole in toluene:acetone, 9:1) spiked with 3-methylindole …..…………………………..S11

Figure S11. GC-MS (TIC) chromatogram of biomass pyrolysis oil……………...……………S11

Figure S12. GC-NCD chromatogram of biomass pyrolysis oil……………………………..…S12

Figure S13. GC-combustion-MS chromatogram for diesel. A) Orange profile corresponds to non-corrected nitrogen and black profile (inset) corresponds to carbon profile obtained in the same analysis (column: BD-EN14103). B) Corrected N-profile…………………………….………S12

Figure S14. GC-MS (TIC) chromatogram for diesel (column: BD-EN14103)……...………..S13

Figure S15. GC-NCD chromatogram for diesel (column: HP-1)……………………….…….…S13

Table S1. Experimental conditions used for the quantitative analysis of N-containing compounds by GC-combustion-MS and GC……………………………….………………………………S14

Table S2. Compound-independent quantification of a mixture of 15 different N-compounds using 2,6-diisopropylaninline as internal standard (Figure S7)………………………………………S15

Table S3. Quantification of total nitrogen content and major families in diesel sample by GC-combustion-MS (Figure S13B) compared with the reference value and GC-NCD…………..S15

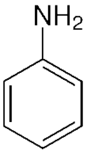<br>Aniline<br>(A, 99.5 %)	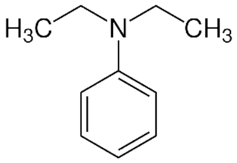<br>N,N-diethylaniline<br>(DEA, 99 %)	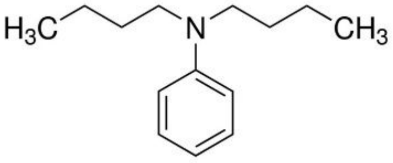<br>N,N-dibutylaniline<br>(DBA, 99 %)
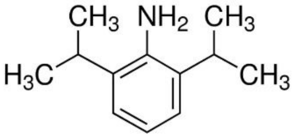<br>2,6-Diisopropylaniline<br>(DPA, 100 %)	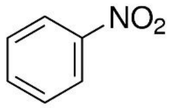<br>Nitrobenzene<br>(NBz, 99 %)	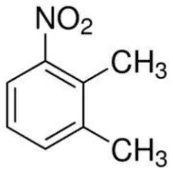<br>1,2-Dimethyl-3-nitrobenzene<br>(DMNBz, 97 %)
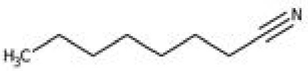<br>Caprylonitrilo<br>(HpCN, 99 %)	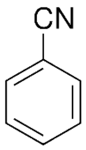<br>Benzonitrile<br>(BN, 99.9 %)	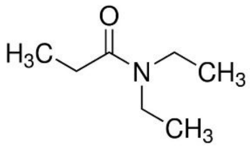<br>N,N-diethylpropionamide<br>(DEPA, 99 %)
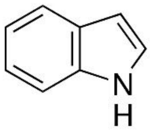<br>Indole<br>(I, 99 %)	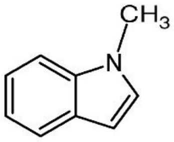<br>1-Methylindole<br>(1MI, 97 %)	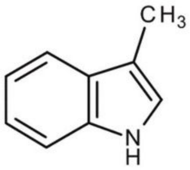<br>3-Methylindole<br>(3MI, 98 %)
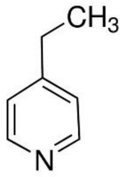<br>4-Ethylpyridine<br>(4EPy, 98 %)	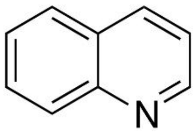<br>Quinoleine<br>(Q, 98 %)	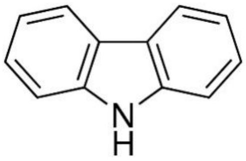<br>Carbazole<br>(C, 95 %)
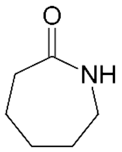<br>Caprolactam<br>(CAP, 100 %)		

Figure S1. List, chemical structure, and purity of the N-containing compounds used.

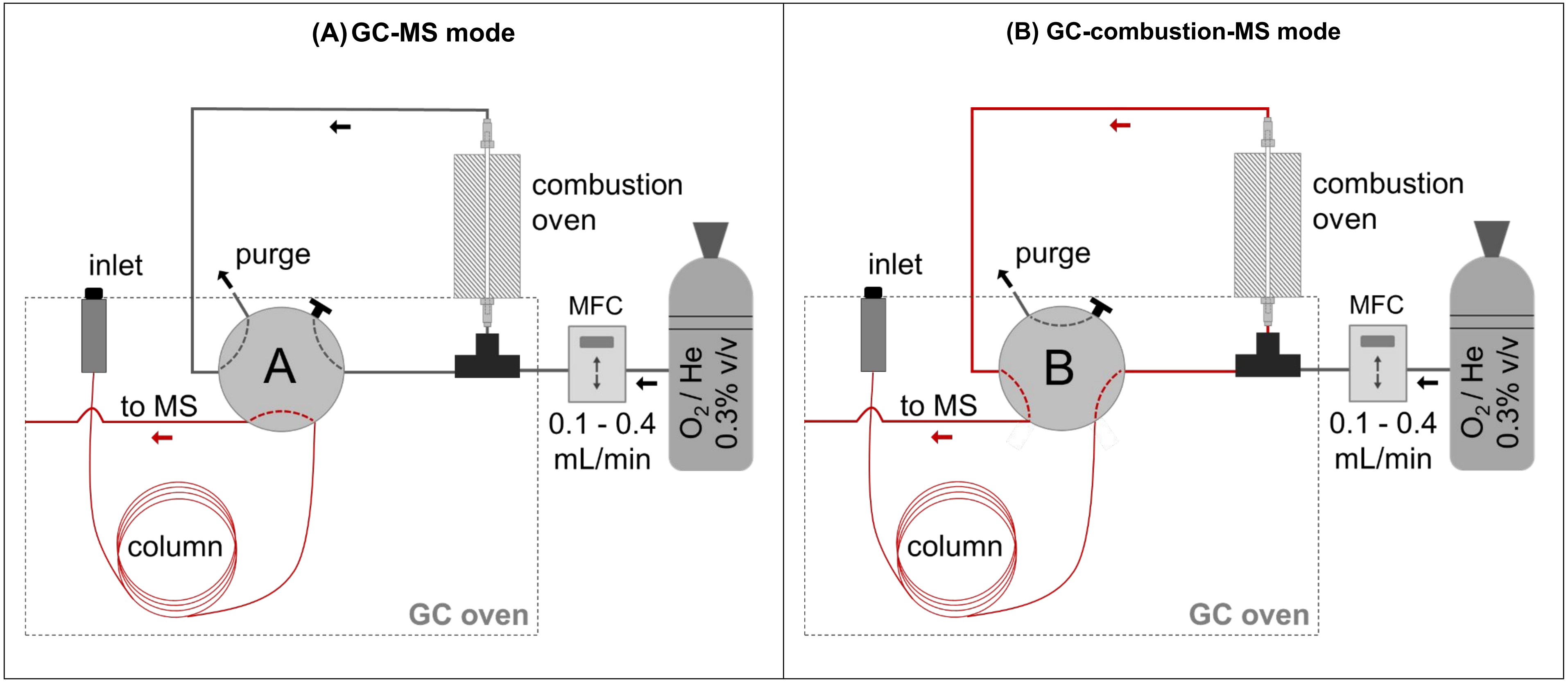

Figure S2. Scheme of the six-way valve and its connections within GC-combustion-MS **prototype #1**. Position A (GC-MS mode): GC effluent is directly sent to the MS. Position B (GC-combustion-MS mode): GC effluent is first mixed online with the O_2/He combustion gas and brought to the combustion furnace before being directed to the MS.

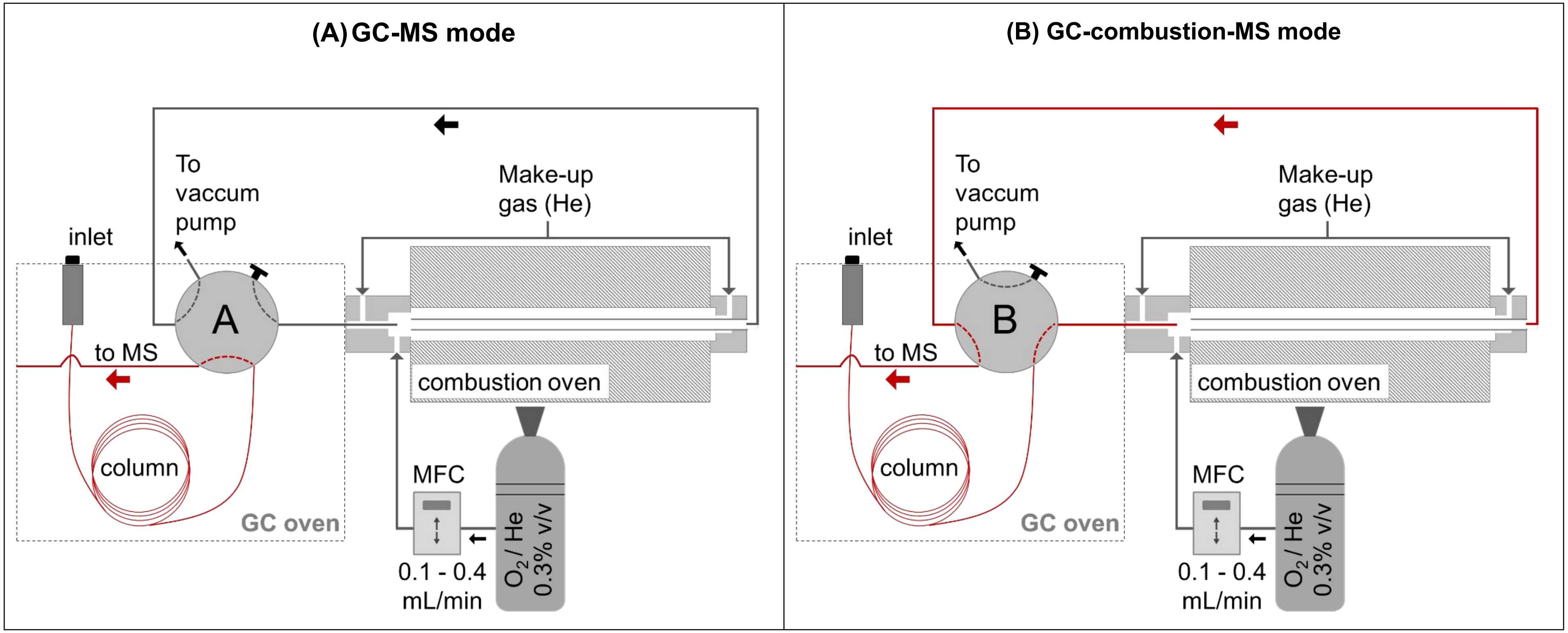

Figure S3. Scheme of the six-way valve and its connections within GC-combustion-MS **prototype #2**. Position A (GC-MS mode): GC effluent is directly sent to the MS. Position B (GC-combustion-MS mode): GC effluent is first mixed online with the O_2/He combustion gas and the He makeup-flow and finally brought to the combustion furnace before being directed to the MS.

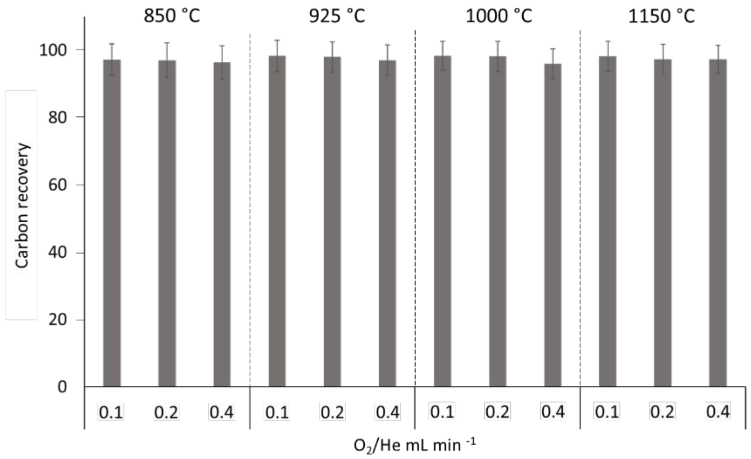

Figure S4. Average of the recoveries for heptadecane (C17) and eight N-containing compounds (DEA, DBA, Q, 1MI, DPA, I, 3MI, see Figure S1) obtained from the CO_2 signals (m/z 44) under different combustion oven temperatures (850, 925, 100 and 1150 °C) and O_2/He flows (0.1, 0.2 and 0.4 mL min^{-1}) and using prototype #1 and pentadecane (C15) as internal standard. Error bars correspond to one standard deviation (n=9).

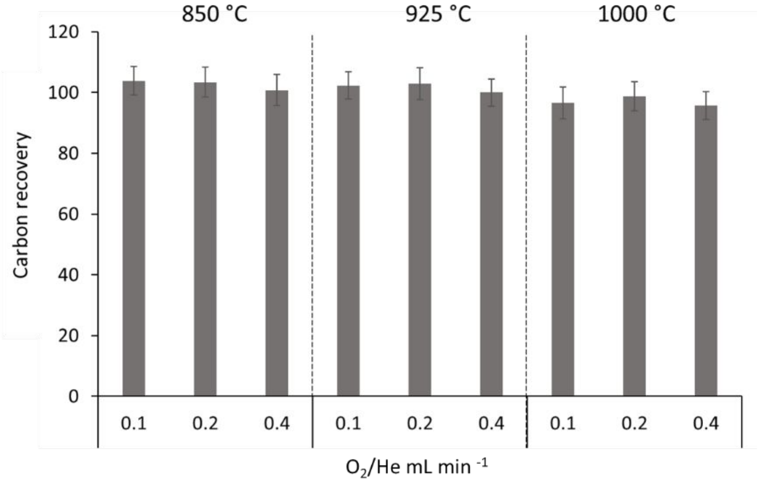

Figure S5. Average of the recoveries for heptadecane (C17) and eight N-containing compounds (DEA, DBA, Q, 1MI, DPA, I, 3MI, see Figure S1) obtained from the CO2 signals (m/z 44) under different combustion oven temperatures (850, 925, and 1000 °C) and O_2/He flows (0.1, 0.2 and 0.4 mL min^{-1}) and using prototype #2 and pentadecane (C15) as internal standard. Error bars correspond to one standard deviation (n=9).

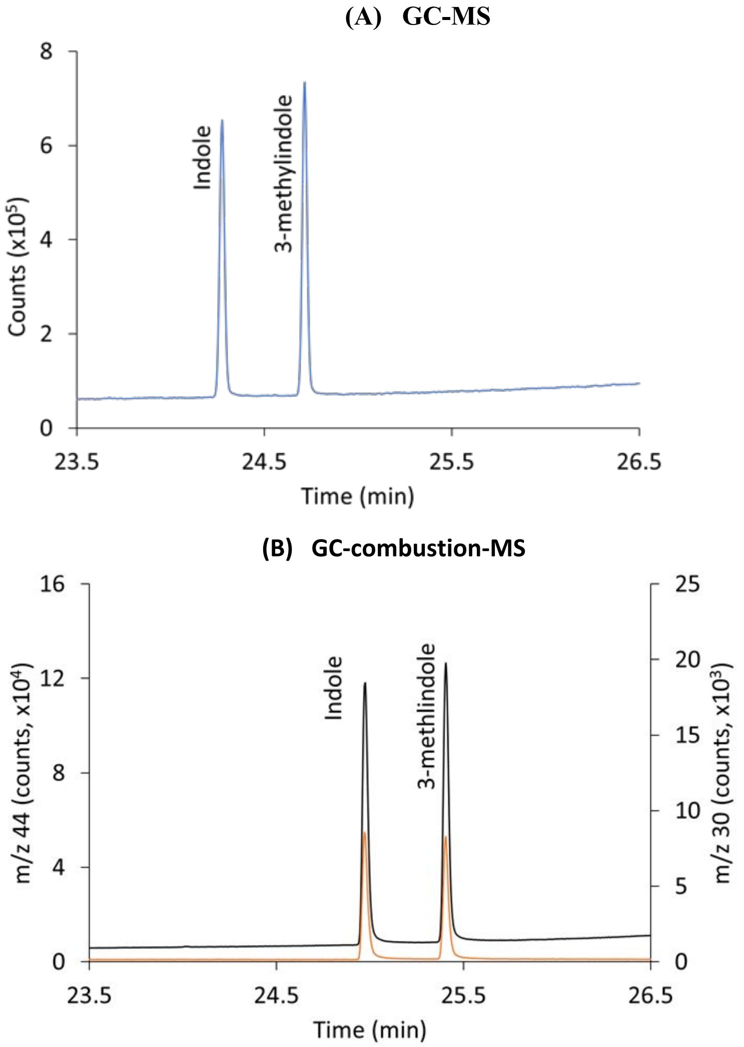

Figure S6. Peak shape comparison of two N-containing compounds, indole (2.1 µg compound/g; 1.7 µg C/g; 0.25 µg N/g) and 3-methylindole (2.2 µg compound/g; 1.8 µg C/g; 0.23 µg N/g) analyzed under: **(A)** GC-MS mode; **(B)** GC-combustion-MS mode (prototype #2) with universal C detection (m/z 44, black) and N-selective detection (m/z 30, orange).

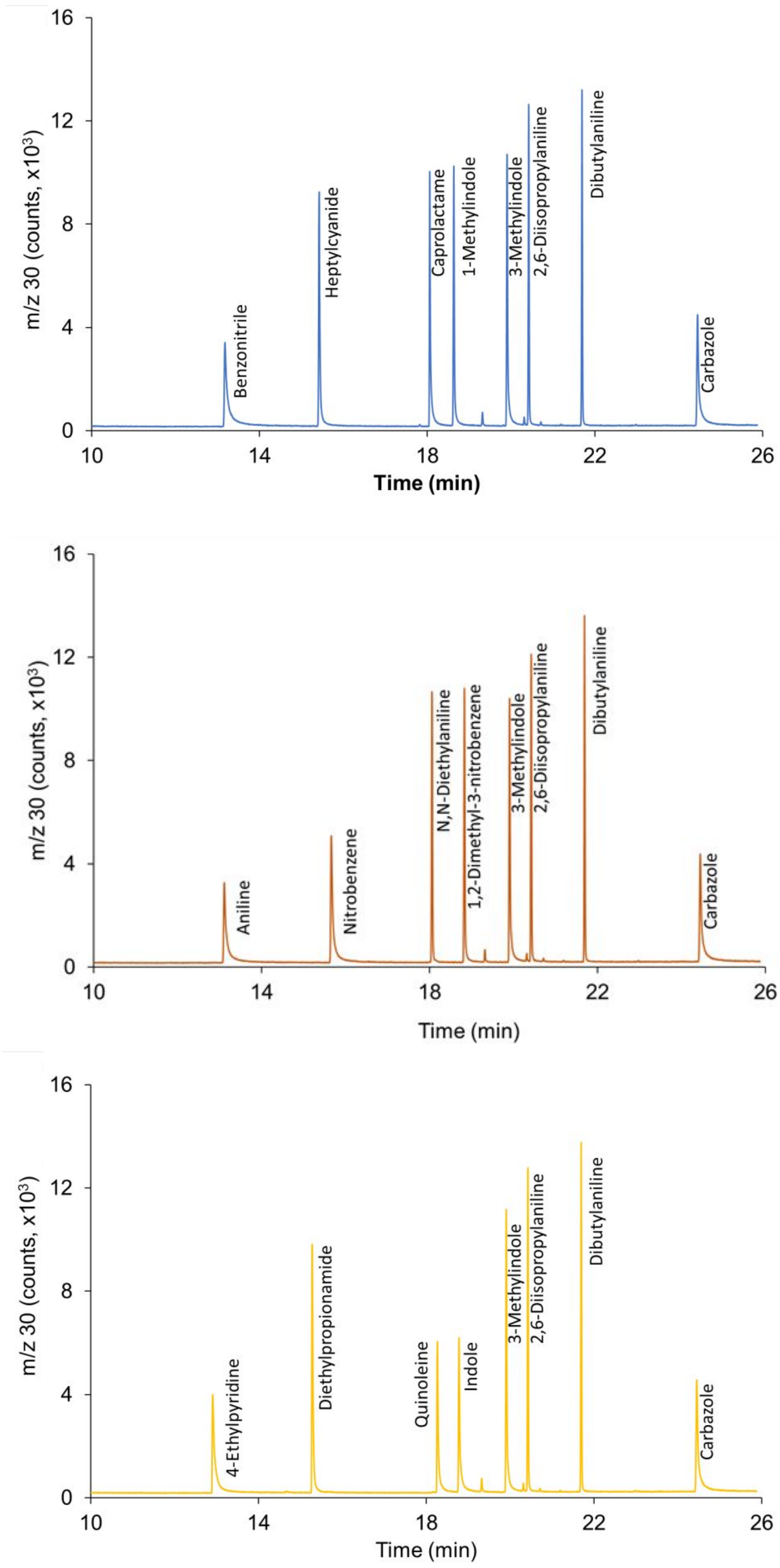

Figure S7. GC-combustion-MS chromatograms (prototype #1) of the extended mixtures of nitrogen-containing compounds. Concentrations in μg N·g^{-1} are: 4EPy=0.61, DEPA=0.65, Q=0.53, I=0.55, 3MI=0.72, DPA=0.59, DBA=0.65, C=0.62, A=0.57, NBz=0.63, DEA=0.56, DMNBz=0.55, BN=0.72, HpCN=0.75, CAP=0.65, 1MI=0.64.

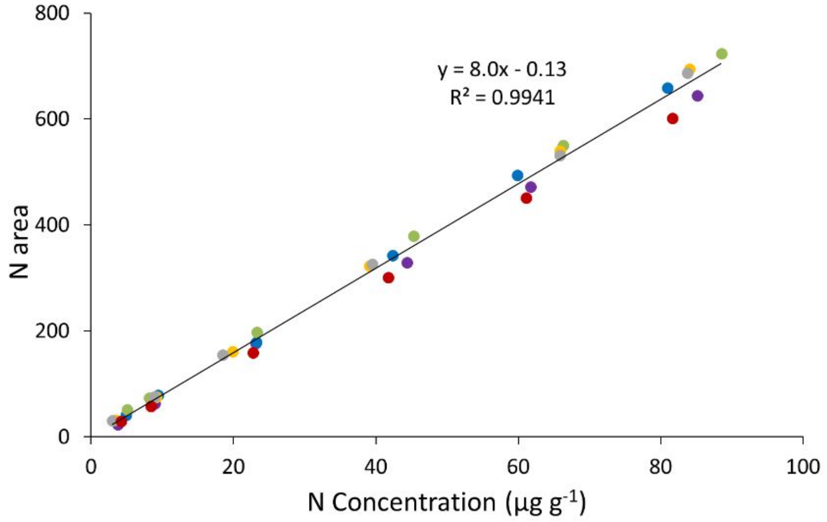

Figure S8. Multispecies generic calibration curve obtained using GC-NCD for six N-compounds in a range of concentrations up to 90 µg N g^{-1}. Color code: Propionitrile (violet), 4-ethylpyridine (blue), Aniline (green), Quinoleine (yellow) Indole (grey), Carbazole (red)

A)

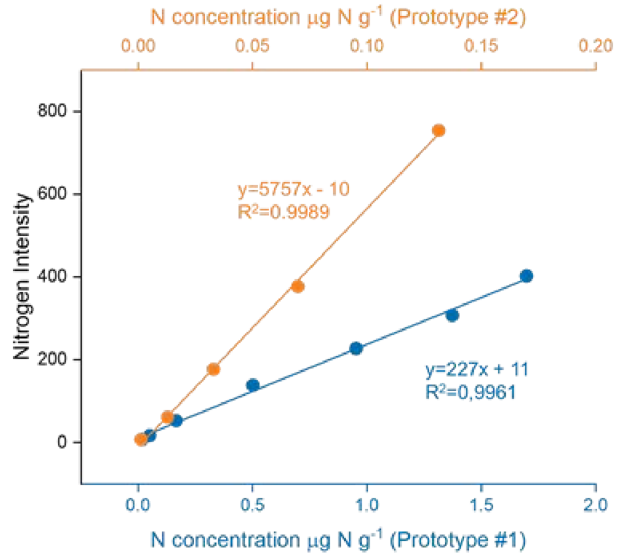

B)

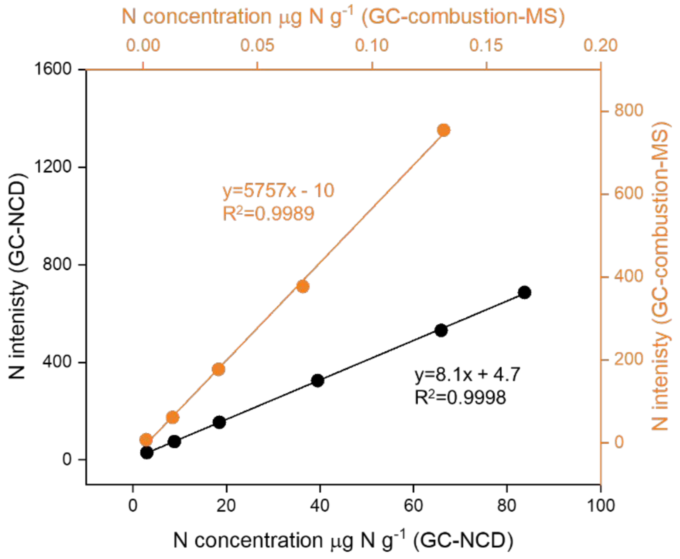

Figure S9. A) Calibration plots obtained for indole using prototype #1 (blue) and #2 (orange). 25-fold increase in sensitivity (slope) was observed for prototype #2. **B)** Calibration plots obtained for indole using prototype #2 (orange) and GC-NCD (black). A 700-fold difference was observed between both systems, what matches well with the difference observed in the limits of detection obtained (0.02 pg of N for GC-combustion-MS vs 2-20 pg N for GC-NCD). Please note that individual chromatograms (raw data, csv files) for the three instruments (prototypes #1 and #2 and NCD) were processed (peak integration) using the same software (OriginPro). In addition, different X-axis (N-concentration) are used for each instrument to allow comparison.

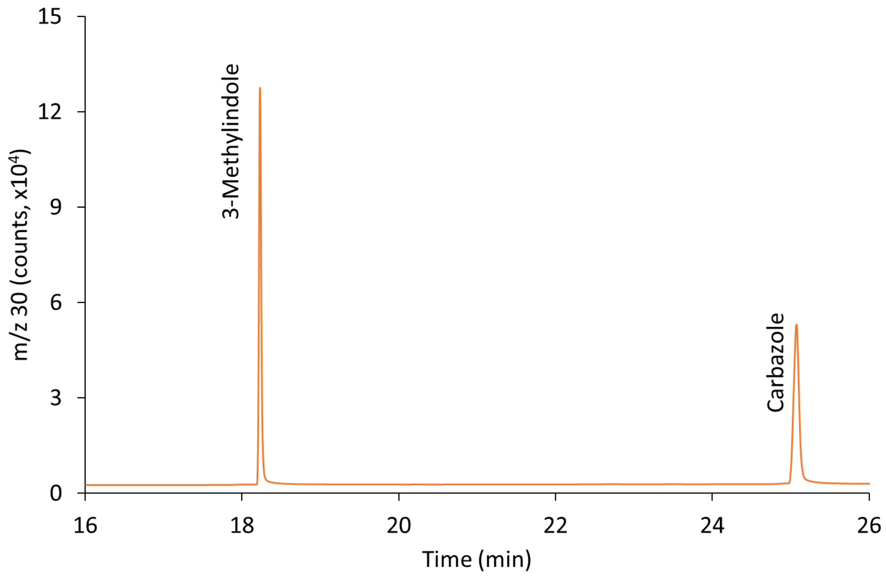

Figure S10. GC-combustion-MS chromatogram of the aliquot of the CRM D-4629-91-HB-CON (carbazole in toluene:acetone, 9:1) spiked with 3-methylindole (c.a. 0.5 µg N g^{-1}) as internal standard.

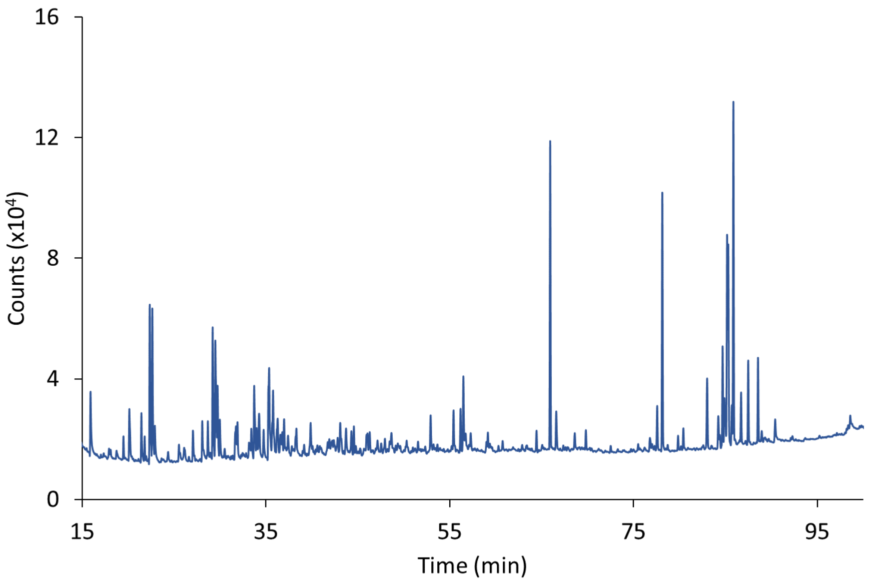

Figure S11. GC-MS (TIC) chromatogram of biomass pyrolysis oil.

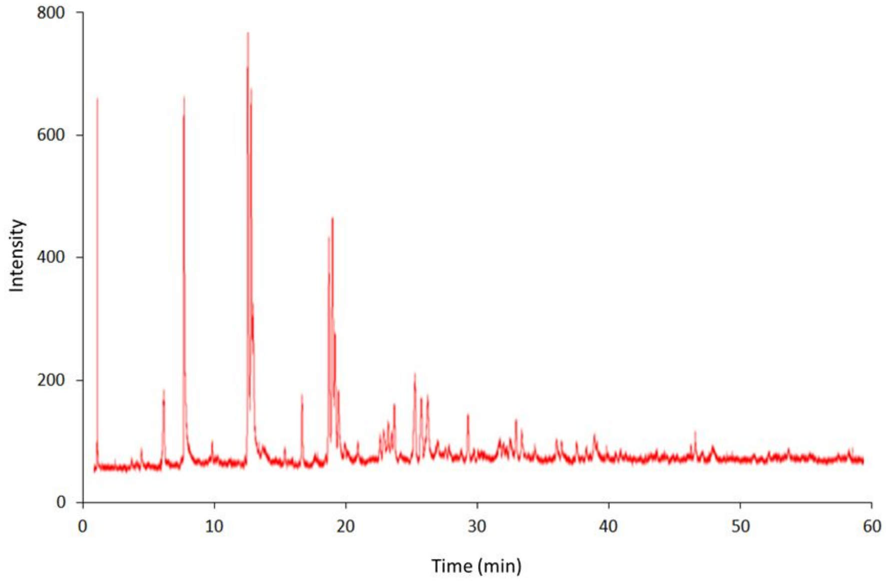

Figure S12. GC-NCD chromatogram of biomass pyrolysis oil.

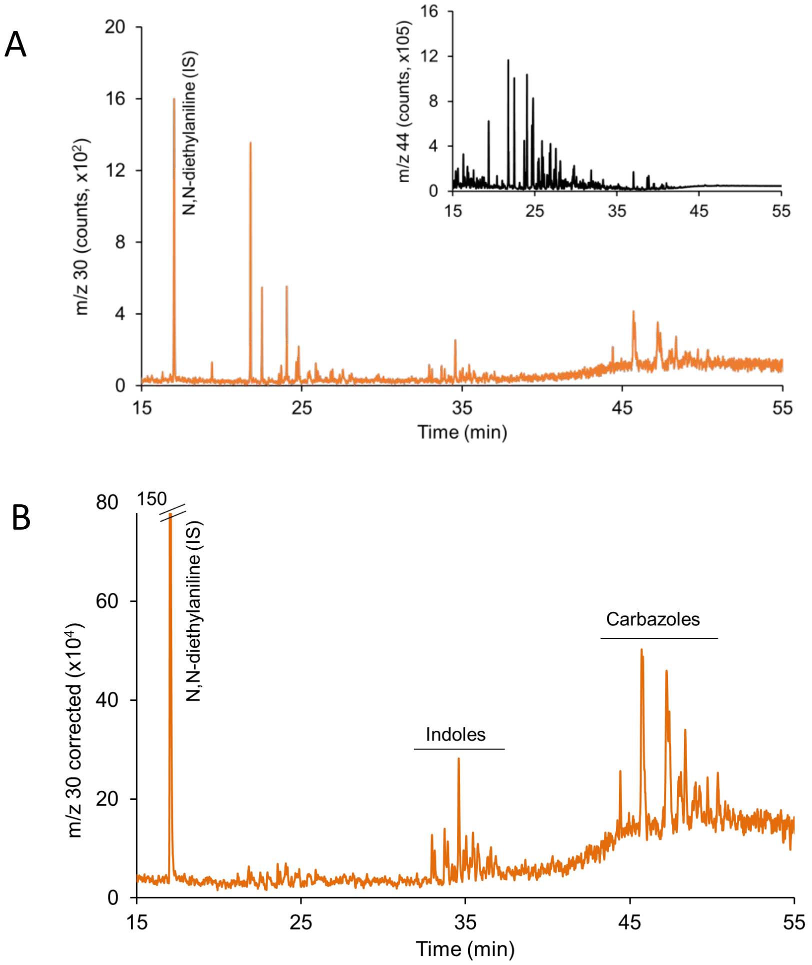

Figure S13. GC-combustion-MS chromatogram for diesel. A) Orange profile corresponds to non-corrected nitrogen and black profile (inset) corresponds to carbon profile obtained in the same analysis (column: BD-EN14103). B) Corrected N-profile

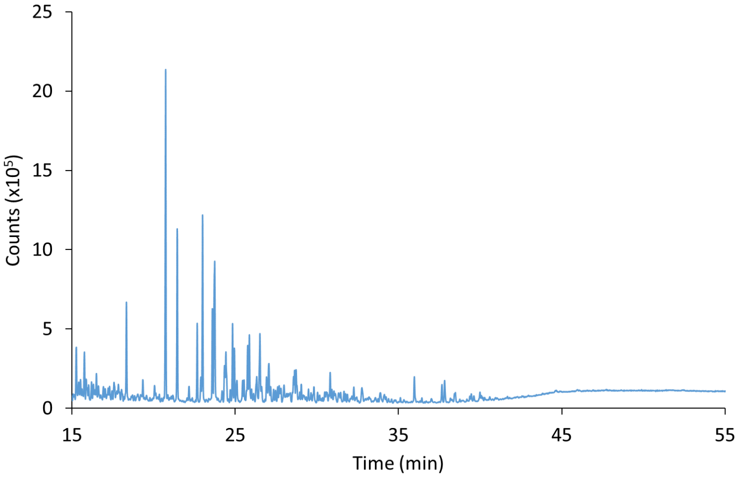

Figure S14. GC-MS (TIC) chromatogram for diesel (column: BD-EN14103)

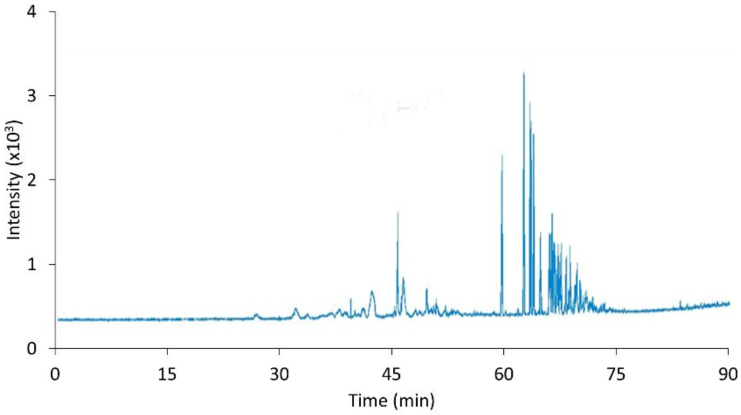

Figure S15. GC-NCD chromatogram for diesel (column: HP-1)

Table S1. Experimental conditions used for the quantitative analysis of N-containing compounds by GC-combustion-MS and GC

Inlet temperature	250 ºC
Injection mode	splitless
Injection volume	1 µL
Column	BD-EN14103 (30 m x 0.32 m x 0.25 µm)<br>HP1-MS (50 m x 0.2 mm x 0.5 µm)
Carrier gas	He (1.5 mL/min)
GC Oven temperature	Standards: 45 ºC (3 min) - 15 ºC/min to 100 (5 min) - 15 ºC/min to 250 (5 min)<br>Diesel: 45 ºC (3 min) - 5 ºC/min to 250 ºC (20 min)<br>Biomass Pyrolysis Oil: 40 ºC (3 min) – 2 ºC/min to 250 ºC (20 min)
Acquisition mode	SIM: m/z 28, m/z 30 and m/z 44
Combustion oven temperature	850-1150 ºC
O_2/He flow	0.1-0.4 mL/min

Additional information of the columns used in the GC×GC-QqQ MS experiments:

- **First dimension:** SLB-5ms (20 m × 0.18 mm ID × 0.18 µm df), silphenylene polymer virtually equivalent in polarity to poly (5% diphenyl/95% methyl siloxane).
- **Second dimension:** SLB-35 (5 m × 0.32 mm ID × 0.25 µm df), bonded and highly crosslinked; proprietary polymer virtually equivalent in polarity to poly (35% diphenyl/65% dimethyl siloxane).

Table S2. Compound-independent quantification of a mixture of 15 different N-compounds using 2,6-diisopropylaninline as internal standard (Figure S7). Added and found N-concentrations and corresponding recoveries (%) are given. Uncertainty corresponds to 2 standard deviations (n=3).

Compound	Added (μg N g^{-1})	Found (μg N g^{-1})	Recovery (%)
4-Ethylpyridine	0.61	0.55 ± 0.04	90 ± 6
N,N-Diethylpropionamide	0.65	0.68 ± 0.01	105 ± 2
Quinoleine	0.53	0.47 ± 0.03	89 ± 6
Indole	0.55	0.49 ± 0.03	90 ± 6
Aniline	0.57	0.56 ± 0.03	98 ± 6
Nitrobenzene	0.63	0.64 ± 0.03	102 ± 5
N,N-Diethylaniline	0.56	0.58 ± 0.01	103 ± 3
1,2-Dimethyl-3-nitrobenzene	0.55	0.60 ± 0.02	111 ± 4
Benzonitrile	0.72	0.71 ± 0.04	99 ± 6
Heptylcyanide	0.75	0.72 ± 0.01	96 ± 2
Caprolactam	0.65	0.65 ± 0.01	100 ± 3
1-Methylindole	0.64	0.62 ± 0.02	97 ± 3
3-Methylindole	0.73	0.69 ± 0.04	96 ± 6
2,6-Diisopropylaniline	0.59	IS	IS
N,N-Dibutylaniline	0.65	0.61 ± 0.01	94 ± 2
Carbazole	0.63	0.61 ± 0.04	97 ± 7

Table S3. Quantification of total nitrogen content and major families in diesel sample by GC-combustion-MS (Figure S13B) compared with the reference value and GC-NCD. Uncertainty corresponds to 2 standard deviations (n=3).

	**μg N g^{-1}**
Total content by chemiluminiscence (ASTM 4629)	**497 ± 10**
GC-combustion-MS:	
Total	**524 ± 22**
Indoles	111 ± 6
Carbazoles	368 ± 25
GC-NCD	**394 ± 42**
